# A Catalyzing Phantom for Reproducible Dynamic Conversion of Hyperpolarized [1-^13^C]-Pyruvate

**DOI:** 10.1371/journal.pone.0071274

**Published:** 2013-08-15

**Authors:** Christopher M. Walker, Jaehyuk Lee, Marc S. Ramirez, Dawid Schellingerhout, Steven Millward, James A. Bankson

**Affiliations:** 1 Department of Imaging Physics, The University of Texas MD Anderson Cancer Center, Houston, Texas, United States of America; 2 Department of Cancer Systems Imaging, The University of Texas MD Anderson Cancer Center, Houston, Texas, United States of America; 3 Department of Diagnostic Radiology, The University of Texas MD Anderson Cancer Center, Houston, Texas, United States of America; University of California, Irvine, United States of America

## Abstract

*In vivo* real time spectroscopic imaging of hyperpolarized ^13^C labeled metabolites shows substantial promise for the assessment of physiological processes that were previously inaccessible. However, reliable and reproducible methods of measurement are necessary to maximize the effectiveness of imaging biomarkers that may one day guide personalized care for diseases such as cancer. Animal models of human disease serve as poor reference standards due to the complexity, heterogeneity, and transient nature of advancing disease. In this study, we describe the reproducible conversion of hyperpolarized [1-^13^C]-pyruvate to [1-^13^C]-lactate using a novel synthetic enzyme phantom system. The rate of reaction can be controlled and tuned to mimic normal or pathologic conditions of varying degree. Variations observed in the use of this phantom compare favorably against within-group variations observed in recent animal studies. This novel phantom system provides crucial capabilities as a reference standard for the optimization, comparison, and certification of quantitative imaging strategies for hyperpolarized tracers.

## Introduction

Quantitative analysis of tissue structure, function, and composition through imaging and imaging biomarkers has the potential to help transform clinical care by facilitating personalized management of diseases such as cancer. Imaging measurements are particularly attractive because they can provide researchers and clinicians with non-invasive, serial views of heterogeneous tumor tissue *in toto,* in stark comparison to the limited number of samples that are practically available through traditional biopsy [1]. In order for imaging biomarkers to provide additional information that positively affects clinical decisions, they must be robust, reproducible, and reflective of relatively specific tissue characteristics. Biomarkers that provide insight into dynamic biological processes are particularly challenging due to the difficulty of establishing a reference truth against which comparisons can be made.

Magnetic resonance imaging (MRI), spectroscopy (MRS), and spectroscopic imaging (MRSI) technologies allow interrogation of anatomic, functional, and molecular characteristics of tissue without the use of ionizing radiation. Traditional MRI, which is fundamentally based on the detection of hydrogen nuclei (^1^H), is sensitive to a wide range of contrast mechanisms that lead to excellent soft-tissue contrast and functional imaging capabilities. Spectroscopic measurements exploit differences in the resonance frequency of nuclei with a nonzero spin, due to changes in the local magnetic field imparted by their molecular environments, to permit quantitative estimation of chemical concentrations *in vivo*. MRSI provides spectroscopic information at points within a slice or volume of interest with varying degrees of spatial and spectral resolution. A vast array of information is available through such measurements, including the ability to generate chemically selective maps of the spatial distribution of key metabolic compounds by ^13^C MRSI.

Unfortunately, magnetic resonance remains a noise-limited measurement and the tremendous clinical potential of MRS/MRSI techniques has not been realized largely due to an intrinsically low signal-to-noise ratio (SNR). SNR limitations are pronounced for MR of ^13^C *in vivo* due to the low natural abundance and MR sensitivity of the ^13^C isotope. Dynamic nuclear polarization (DNP) of ^13^C-enriched tracers enables dramatic increases in the excess spin population and thus the observable ^13^C MR signal [2], [3]. The hyperpolarized state created by DNP has been shown to increase SNR by approximately 10^4^-fold compared to normal thermal equilibrium for select substrates such as [1-^13^C]-pyruvate [3]. This increase in signal has made *in vivo* spectroscopic imaging of hyperpolarized (HP) tracers feasible [4]. However, the signal from HP tracers is non-renewable and short-lived due to intrinsic relaxation mechanisms and signal depletion from necessary radiofrequency (RF) excitations, placing fundamental constraints on the measurement of their spatial and chemical fate. All acquisition protocols reflect a balance of acquisition time, the number and kind of signal excitations, and spatial, spectral, and temporal resolution. A range of data reduction techniques aimed at extracting more information from fewer signal excitations have been employed [5]–[13]. Optimization of acquisition strategies with respect to these unique constraints is ongoing, but assessment of the accuracy and reproducibility of these methods remains a challenge. Repeatable calibration standards would help characterize the accuracy and reproducibility of measurements under various data reduction strategies, a critical step in the translation of these powerful new biomarkers to clinical use.

^13^C-labeled pyruvate is the most studied hyperpolarized tracer to date because of its strong polarization, favorable kinetics, and central role in metabolism. The chemical fate of pyruvate is of particular interest in oncology because many cancers display the ‘Warburg effect’ and metabolize glucose, the precursor of pyruvate, using mechanisms that are less efficient for energy production but are thought to provide other advantages for survival and proliferation of disease [14], [15]. Preservation of nuclear spin state through chemical conversion allows us to observe the spatial distribution of HP-pyruvate and of HP metabolites such as lactate, alanine, and bicarbonate that can only arise through interactions between tracer and the relevant metabolic enzymes. Early studies have shown that a decrease in the conversion rate of HP pyruvate to lactate correlates with response to therapy and tumor grade [4], [16]–[19].

In this work, we demonstrate a phantom system in which the conversion of hyperpolarized pyruvate to lactate can proceed in the controlled, tunable, and reproducible environment of an isolated buffer. Hyperpolarized pyruvate is injected into a vessel containing the reagents that are necessary for reproducible conversion of pyruvate to lactate at a rate that is consistent with in vivo observations. This platform provides a robust tool for evaluation and comparison of spectroscopic and imaging measurements without the added complexity of a biological system. Many sources of variability inherent to the use of HP tracers *in vivo* are eliminated, allowing focused study of controllable parameters in order to maximize SNR and minimize measurement errors that can lead to artifacts. The ability to carry out these reactions at a known rate over a predetermined spatial distribution will significantly accelerate the optimization and validation of instrumentation and strategies for efficient signal acquisition, reconstruction, and analysis. This work focuses on [1-^13^C]-pyruvate due to its progress in clinical trials and near-term potential for clinical use [4]. Similar phantoms and calibration standards can be developed for other HP tracers, employing alternative enzyme systems.

## Methods

Pyruvate is specifically converted into lactate by the enzyme lactate dehydrogenase (LDH) and the reduced form of the coenzyme nicotinamide adenine dinucleotide (NADH):
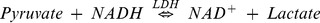
(1)


This ordered ternary complex can be modeled using classical enzyme kinetics [20]–[23] to derive velocities (Mol/s) of the reaction as a function of constituent concentrations. The Gibbs free energy for this reaction is large (ΔG’^0^ = −25.1 kJ/mol), strongly favoring the forward reaction and lactate production; under normal physiological conditions, these reagents are involved in multiple cellular processes that modify their cytosolic concentration [24].

Under normal conditions for MRSI of HP lactate and pyruvate, the signal from ^13^C at thermal equilibrium is below the threshold of detectability, and only polarized labels can be observed. The rate of chemical conversion for HP tracers depends on the velocities of the chemical reaction and the likelihood that reactants are polarized:
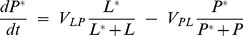
(2)

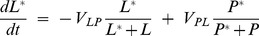
(3)


Here, V_PL_ denotes the forward velocity of the reaction, for conversion of pyruvate to lactate, and V_LP_ represents the reverse reaction; *L* is the concentration of unpolarized lactate, P represents the concentration of unpolarized pyruvate, and the concentration of the polarized spin pools for each metabolite are indicated by asterisk. Witney et. al. [20] presented a model to describe the net conversion of pyruvate to lactate as well as the forward velocity of HP label exchange.

### A. Modeling

Equations 2–3 can be modified to account for spin-lattice (T_1_) relaxation and losses due to signal excitation:
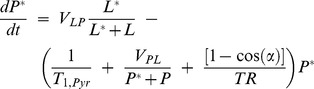
(4)

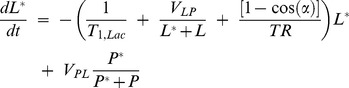
(5)


Where *T_1,pyr_* and *T_1,lac_* are the spin lattice relaxation time constants for pyruvate and lactate respectively, α is the flip angle of the RF excitation pulse, and *TR* is the repetition time. Using the models outline by Witney et. al. [20] and equations 4 and 5, a finite-difference approach was implemented in Matlab (The Mathworks, Natick, MA) to calculate conversion and exchange as a function of the total concentrations of pyruvate, lactate, LDH, NADH, and NAD+, and the percent polarization of the tracers. This model was used to determine phantom reagent concentrations that would reduce reaction rate variability.

Kinetic models with multiple compartments can be used to determine the flux of hyperpolarized tracers. To account for the conditions of an *in vivo* environment, models with two to six chemical and physical pools have been proposed [25]–[27]. One clear advantage of a single enzyme phantom is that the minimal kinetic model, employing only two chemical and no spatially separate pools within a voxel, can be applied without simplifying assumptions of unknown effect. Briefly, the equilibrium between pyruvate (Pyr) and lactate (Lac) can be described by:
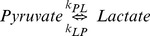
(6)


Where k_PL_ and k_LP_ are the unidirectional rate constants of the LDH catalyzed conversions. By defining 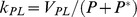 and 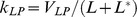, and recognizing that observations are samples of the longitudinal magnetization of each pool, the two-compartment model can be expressed in a more suitable form [25], [28], [29]:
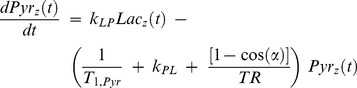
(7)

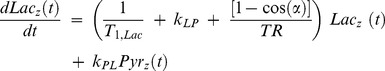
(8)


In contrast to the isolated phantom system where reagents can be accurately quantified, in vivo measurements based on this model reflect apparent rate constants due to the uncertainties in the cellular concentration of unlabeled reagents. Leveraging known reagent concentrations, time series of hyperpolarized pyruvate and lactate signal levels measured as described below were fit to this model using custom software developed in Matlab (The Mathworks, Natick, MA). Rate constants were determined by fitting dynamic tracer curves to equations 7 and 8 in the least squares sense, as previously described [25], [28], [29].

### B. Phantom Preparation

**Figure 1:**
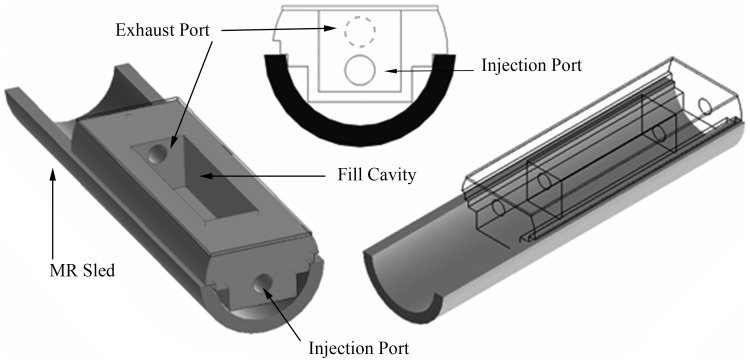
A schematic view of the dynamic chemical phantom structure. The injection and exhaust ports were fitted with catheters to facilitate rapid mixture of reagents at isocenter. A thin acrylic sheet was attached to the top to seal the fill cavity. This top could be removed to allow cleaning after injection. The phantom rested on a sled that allowed convenient removal and insertion of the phantom and included warm circulating water to maintain constant temperature.

Phantom concentrations were qualitatively optimized to reduce reaction rate sensitivity to variabilities in the concentrations of its components and ensure the reaction ran to completion before hyperpolarized signal decayed below threshold of detectability and as consistent with previous in vivo observations. Special consideration was given to reduce sensitivity due to pyruvate concentration and LDH activity as these were assumed to be the least reproducible characteristics of the phantom system. A custom phantom container was machined out of cylindrical Ultem resin stock and fitted with a 1 m polyethylene 3.175 mm diameter catheter (Coilhose Pneumatics, East Brunswick, NJ) for remote injection into the cavity when located at the isocenter of the magnet. The rectangular cavity was 1×1×3 cm with the injection catheter connecting to the front as seen in Figure 1. This structure was used for assessment of the enzymatic phantom using dynamic spectroscopy. To test the feasibility for such a reaction to be conducted inside a phantom with spatial details necessary to validate spectroscopic imaging sequences, a standard MRI quality assurance phantom (Aufloesung 30 KIT G, model 1P T58930; Bruker Biospin MRI, Ettlingen, Germany) was drained and refilled with the catalytic mixture described below.

All reagents save pyruvate were thawed from aliquoted fresh solutions stored at −80°C and mixed in a 5 mL syringe shortly before completion of the polarizing process as described below. Once polarization was complete, HP pyruvate was injected into the phantom and followed by the enzyme substrate mixture to fill the phantom cavity. Nominal final concentrations were 2 mM hyperpolarized ^13^C-Pyruvate, 40 mM Lactate, 3.92 U/mL LDH (Worthington Biochemical Corp., Lakewood, NJ), and 4 mM Β-NADH (Sigma-Aldrich Corp., St. Louis, MO) in the Tris buffer (81.3 mM trisma preset crystals pH 7.2, 203.3 mM NaCl) (Sigma-Aldrich). This specific configuration places the phantom far from chemical equilibrium, rendering the reverse reaction (*V_LP_, k_LP_*) negligible throughout. The phantom was held at 28°C with a final pH of 7.2 and 3-mL final volume. UV spectrophotometry indicates that enzyme activity remains unchanged for at least two hours at room temperature.

### C. Hyperpolarization

13 mg of [1-^13^C]-pyruvic acid (Sigma-Aldrich) with 15 mM OX063 (GE Healthcare, Fairfield, CT) and 0.0.775 mM Prohance (Bracco Diagnostics) were hyperpolarized in a HyperSense DNP system (Oxford Instruments) as described previously [3], [30]. The sample was dissolved in 4 mL of buffer consisting of 40 mM Trizma pre-set crystals (pH 7.6), 40 mM NaOH, 50 mM NaCl, and 0.1 mg/L EDTA. Once the dissolution process was complete, 0.15 mL of HP [1-^13^C]pyruvate (nominally 30% polarization) was drawn into a syringe for injection into the phantom.

### D. Dynamic Spectroscopy

Dynamic spectroscopy was performed on a 7-T/30-cm Biospec System (Bruker Biospin Corp., Billerica, MA) using B-GA12 gradients (120-mm inner diameter (ID), G_max_ = 400 mT/m) and a dual-tuned ^1^H/^13^C volume coil (72-mm ID, Bruker Biospin MRI). Dynamic ^13^C spectra were acquired with a 2.5 kHz bandwidth, 4098 points, 10° excitation using a 1 ms Gaussian pulse, 2-sec repetition time (TR), with 60 repetitions over a 3-min scan time beginning at dissolution and triggered by the HyperSense system. To evaluate performance and repeatability, the measurement was repeated seven times using identical reagent concentration.

Signal from each metabolite was determined by calculating the area of the Lorentzian line shape that fit most closely in the mean square sense. Dynamic signal curves for each tracer were integrated in time to estimate total signal from pyruvate and lactate. Their sum represents the total signal observed from all HP ^13^C-labeled metabolites in this phantom system. Signal amplitudes were normalized to account for variations in the amount of polarized pyruvate present at the onset of scanning. Three quantitative parameters were used to characterize the reaction rate for each measurement: total lactate signal normalized to total pyruvate, or to the sum of total pyruvate and lactate signal, and *k_PL_*, the forward rate constant for the two compartment model described by equations (7) and (8).

### E. Spectroscopic Imaging

To demonstrate the usefulness of the enzyme phantom on evaluating spatial sequence performance, a 10-mL standard imaging phantom was drained and fitted with the same injection catheter described above. A slightly lower concentration of NADH (2 mM) was used in an otherwise identical mixture due to the increased phantom volume. A custom built dual-tuned ^1^H/^13^C linear birdcage coil with a 35 mm ID was used in conjunction with B-G6 gradients (60-mm ID, G_max_ = 1000 mT/m, Bruker Biospin Corp.). The phantom was scanned with a radial echo planar spectral imaging (EPSI) sequence (unpublished). This was a single shot acquisition that expunged the entire hyperpolarized signal to acquire a single set of spectroscopic imaging data. The acquisition was started ∼40 seconds after all components were combined in the phantom, and data was acquired with a repetition time of 60 ms, initial echo time of 5.5 ms, and 1.3 ms echo spacing to form a 32 point echo train. A variable flip angle was used to maintain approximately equal sampling of longitudinal magnetization [31]. Spectral bandwidth was 23.8 kHz with a 744 Hz or 9.85 ppm spectral width. Fifty spatial projections were taken with 32 readout points over a 4 cm by 4 cm field of view and a 2 cm slice thickness.

## Results

**Figure 2:**
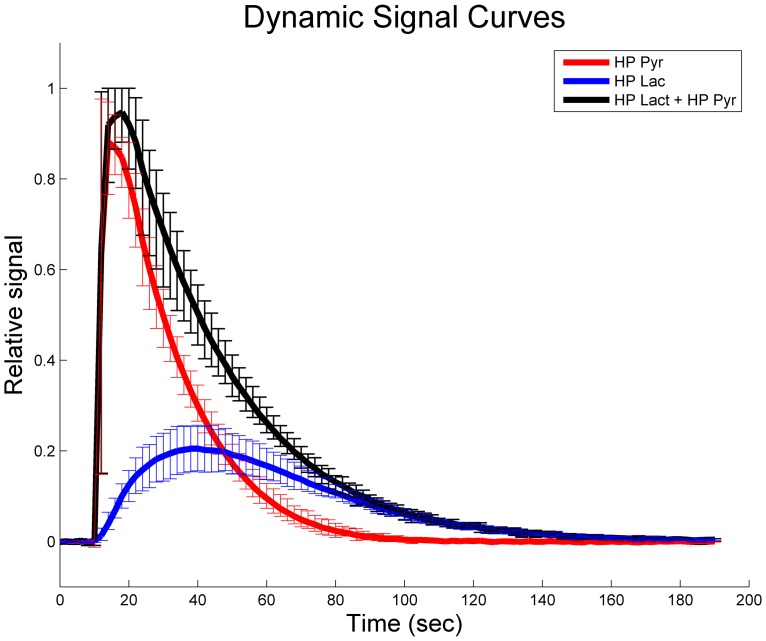
Dynamic signal evolution across (N = 7) injections. The mean signal for lactate and pyruvate, normalized to peak carbon signal for each injection, are displayed with error bars that indicate the minimum and maximum values at each time over all injections. Total HP ^13^C was estimated by summing signal from HP ^13^C Lactate and HP ^13^C Pyruvate. The average linewidth for pyruvate and lactate peaks were 19±5 Hz and 17±5 Hz, respectively.

Isolating the enzymes in a system where initial reagent and product concentrations can be controlled allows the exchange rate to be specifically tuned and modeled without interference from other confounding biological processes. Simulations suggested that a phantom system containing 2 mM hyperpolarized [1-^13^C]-pyruvate, 40 mM lactate, 3.92 U/mL LDH, and 4 mM Β-NADH would progress at a rate that is consistent with *in vivo* observations [30] with reduced sensitivity to experimental variations. The phantom system, shown in Figure 1, was assembled and tested (N = 7 replicates), demonstrating reproducible conversion of hyperpolarized tracer as summarized in Figure 2. After a brief delay between start of data acquisition and injection of the polarized tracer, the pyruvate signal peaks quickly as it fills the chamber. A small frequency shift of 0.2 ppm, likely due to changes in susceptibility in the chamber, was observed during injection. Pyruvate signal decays due to relaxation, signal excitation, and chemical conversion to undetectable levels in less than two minutes. The lactate signal rises until the growth of the HP lactate pool is less than losses due to relaxation and signal excitation, at which point it similarly decays. The coefficient of variation for common measures of this reaction, summarized in Table 1, compare favorably to within-group variations reported in 9 recent animal studies [16]–[19], [32]–[37] as summarized in Table 2.

**Table 1:** The mean, standard deviation and coefficient of variation for all repetitions (N = 7) of the dynamic phantom.

Parameter	Mean	Standard Deviation	Coefficient of Variation
Lac/(Lac+Pyr)	0.391	0.048	12.3%
Lac/Pyr	0.652	0.124	19.0%
*k_PL_*	0.020	0.0038	19.0%

Lac and Pyr refer to the total volume under the spectral and temporal curves for each tracer; *k_PL_* is the forward reaction rate (see Equations 6–8).

**Table 2:** Survey of HP parameter variation in recent animal studies.

Reference	Location	Disease	Parameter	Number of animals	Average within group error
Albers [16]	Prostate	Cancer	Metabolite SNR	5,4,3,3	25%
Day [17]	Subcutaneous	Lymphoma	k_pl_	8	17%
Laustsen [41]	Kidney	Diabetes	Lac/(Total 13C Signal)	10,6	40%
Thind [32]	Thorax	Radiation Injury	Lac/Pyr	6,4,5	26%
Bohndiek [18]	Subcutaneous	Colorectal Cancer	Lac/Pyr	Not Available	24%
Park [19]	Brain	Glioblastoma	Lac/Pyr	7,9	54%
Bohndiek [35]	Subcutaneous	Lymphoma	k_pl_	10,7,7	37%
Matsumoto [36]	Subcutaneous	Squamous Cell Carc.	Lac/(Total 13C Signal)	5,4	12%
Lau [37]	Heart	Normal	Lac/(Total 13C Signal)	11,6	28%
Average					29%

**Figure 3:**
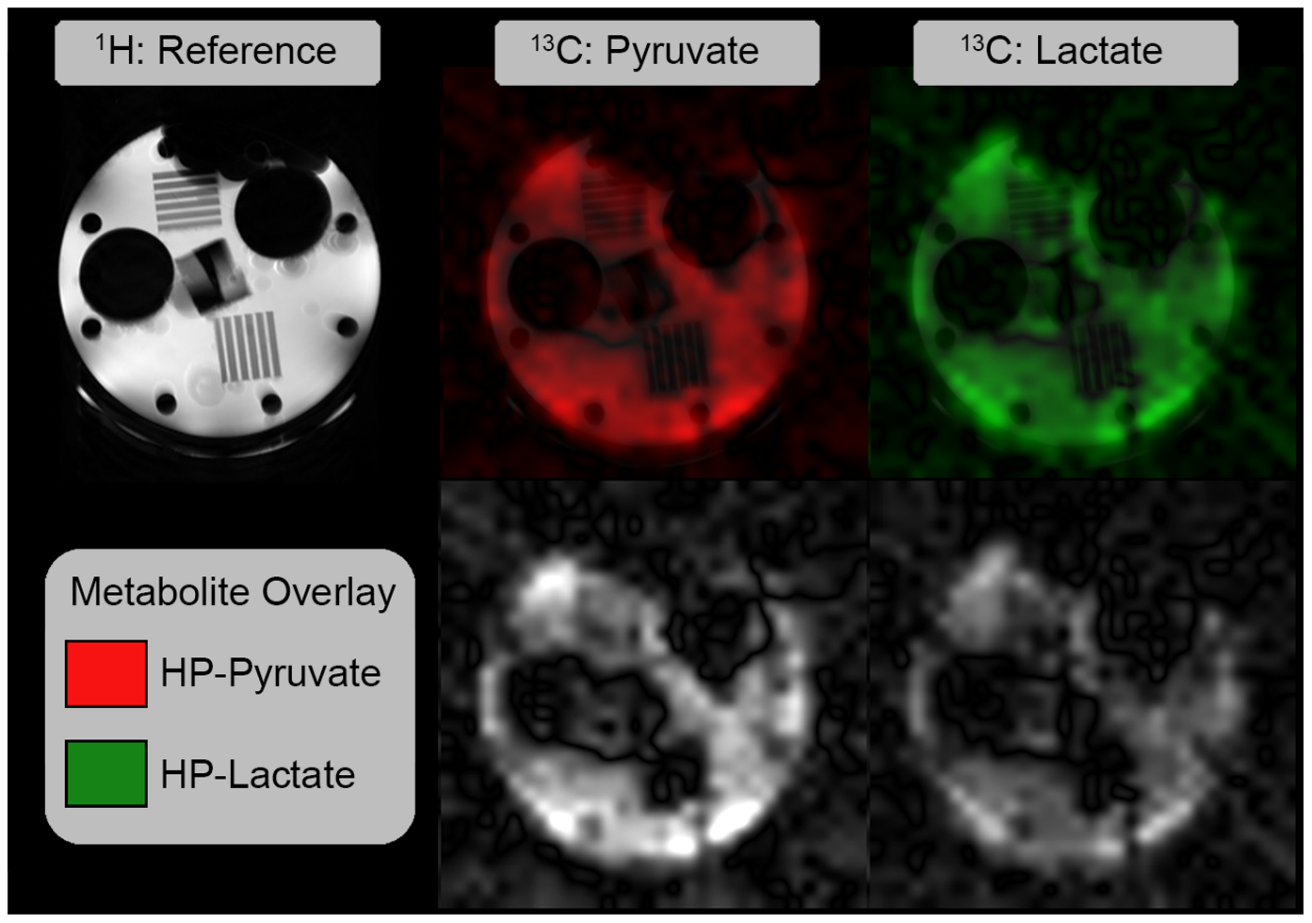
Spectroscopic images of the reaction carried out in a standard imaging phantom. Proton imaging (top left) shows the phantom structure in high resolution. Spectroscopic imaging data acquired using a radial EPSI sequence allows metabolite-specific visualization of tracer distribution (bottom row). Spectroscopic data can be intrinsically registered to high-resolution proton images (top center and right).

Snapshot spectroscopic imaging of the separate imaging phantom shows relatively homogeneous mixture of components and distribution of tracer and metabolite 40 s after initiation of the reaction. Images of HP pyruvate and lactate, alone and in overlay over reference proton images, can be seen in Figure 3. While the resolution of the MRSI sequence is significantly lower than the proton image, it is possible to resolve features within spectroscopic images for both individual metabolites. No significant spatial distortions are seen.

## Discussion

Real time *in vivo* spectroscopic measurement of labeled metabolites and their products is now possible with the integration of key polarizable tracers and carefully designed strategies for encoding, acquisition, reconstruction, and analysis of signals that are observable through magnetic resonance. Imaging the metabolic conversion of pyruvate into lactate shows promise as a clinically useful biomarker for oncology [4]. Hyperpolarized contrast agents comprise a relatively new and rapidly developing field, and research into best practices for signal acquisition, reconstruction, and analysis is ongoing. This dynamic phantom will provide robust, reproducible, and tunable dynamic processes against which experimental strategies can be compared and optimized.

This phantom system provides new capabilities for experimental development and validation with distinct advantages over single-tracer phantoms, static multi-compartment thermal equilibrium phantoms, and in-vivo models. Static phantoms are useful for confirming some functionality, such as the signal-to-noise ratio and spatial resolution associated with specific measurement settings, but do not create the dynamic conditions that could lead to artifacts in reconstruction algorithms that are based on the assumption of a stationary target. Assessment using *in vivo* models is challenging because of biological heterogeneity and the evolution of target processes in diseases such as cancer that can progress rapidly and increase within-group variations in a matter of days. With this platform, acquisitions can be readily repeated, at arbitrary intervals, to extract statistical measures of image quality.

To our knowledge, this is the first reproducibility study of a phantom system that provides controlled dynamic evolution of a chemical process that is observable by magnetic resonance using hyperpolarized tracers. This system catalyzes the final step in aerobic glycolysis, the conversion of pyruvate into lactate, without the need for animal subjects, human subjects, or cell suspensions that can increase the cost and the variability of technical measurements. The 12.3–19% variation that we observed is a result of many factors. LDH is sensitive to a range of experimental circumstances [38]; small variations in temperature, pH, or storage conditions can affect enzyme activity and therefore the rate of the reaction. In this pilot work, the injection of a small amount of hyperpolarized pyruvate was performed by hand, potentially leading to unnecessarily high variations in the end concentration of pyruvate. In future investigations, this error will be reduced by utilizing automated injections and a refined mixture and delivery system that reduces local fluctuations in the composition of the system [39].

The compatibility of this concept with a wide range of potential phantom structures provides clear opportunities for future use. Multiple compartments can easily be incorporated into new or existing phantom structures, and reaction rates in distinct regions can be tuned by varying reagent concentrations to simulate different tissues or disease states in parallel. In addition, a separate chamber that is free of enzyme would allow normalization to account for variations in tracer polarization. Because the spatial characteristics of such a phantom would be known *a priori*, rigorous evaluation and optimization of data encoding, acquisition, and reconstruction algorithms is possible. This is especially important when considering data reduction strategies that are designed to address key limitations in the measurement of hyperpolarized tracers but blur traditional definitions of spatial and temporal resolution in the observation of dynamic processes. Such a platform would be ideal for exploration of thresholds for detectability of pathologies that may not be evident in ^1^H MRI, early testing of new sequences to ensure preservation of spatial and temporal accuracy, and for quality assurance scans to confirm that similar acquisition, reconstruction, and analysis parameters lead to similar data over time both within and between laboratories and institutions..

Our phantom structure for dynamic spectroscopy (Figure 1) was designed using ultem (χ_V_∼−8.9×10^−6^) to minimize susceptibility differences with water (χ_V_∼−9.03×10^6^) at interfaces that are normal to the static field. However, air (χ_V_∼0.36×10^−6^) on one side of the rectangular volume of the chamber led to approximately 0.2 ppm shift in the resonant frequency of metabolites as the chamber was filled. More complex phantom structures will require careful design in order to minimize frequency shifts due to susceptibility differences that are modified as chambers are filled.

A crucial step in the translation of powerful new imaging technologies into routine preclinical and clinical use is the establishment of well-defined reference standards [40] to provide a common reference against which experimental circumstances can be compared. Such a reference can be used to ensure comparable results across platforms, laboratories, and institutions, and aid in study design and execution. The dynamic single enzyme phantom helps fill this critical need by providing reproducible HP tracer evolution that is independent of complex biological barriers and heterogeneity that cannot be strictly controlled. The physical structure of the phantom can be tailored to more closely approximate preclinical or clinical applications, and the rate of the reaction can be controlled through multiple compartments in a spatially dependent manner to simulate a wide range of disease states. This phantom platform represents a flexible and powerful tool to aid in development, optimization, validation, and certification of techniques, processes, and instrumentation that are crucial to ensure the successful and efficient translation of powerful new imaging capabilities afforded by MRSI of hyperpolarized tracers such as [1-^13^C]-pyruvate.
